# Improvements in blood transfusion management: cross-sectional data analysis from nine hospitals in Zhejiang, China

**DOI:** 10.1186/s12913-018-3673-x

**Published:** 2018-11-14

**Authors:** Yuanyuan Yao, Jun Li, Mingcang Wang, Zhonghua Chen, Weixing Wang, Lipei Lei, Changshun Huang, Ming Yao, Guihua Yuan, Min Yan

**Affiliations:** 1 Department of Anesthesiology, the Second Affiliated Hospital, School of Medicine, Zhejiang University, Hangzhou, 310009 China; 2 The 2nd Affiliated Hospital & Yuying Children’s Hospital of Wenzhou Medical University, Wenzhou, China; 3 Taizhou Hospital of Zhejiang Province, Taizhou, China; 4 Shaoxing People’s Hospital, Shaoxing, China; 5 Huzhou Central Hospital, Huzhou, China; 6 The Central Hospital of Lishui City, Lishui, China; 7 Ningbo First Hospital (Ningbo Hospital of Zhejiang University), Ningbo, China; 8 The First Hospital of Jiaxing, Jiaxing, China; 9 Shaoxing Central Hospital, Shaoxing, China

**Keywords:** Quality improvement, Health policy, Blood transfusion, Patient safety, Surgery

## Abstract

**Background:**

Since 2008, updated perioperative blood management (PoBM) guidelines have been implemented in Zhejiang, China. These guidelines ensure that the limited blood resources meet increasing clinical needs and patient safety requirements. We assessed the effects of implementing updated PoBM guidelines in hospitals in Zhejiang, China.

**Methods:**

We performed a retrospective multicenter study that included adult patients who received blood transfusions during surgical care in the years 2007 and 2011. The volume of allogeneic red blood cells or autologous blood transfusions (cell salvage and acute normovolemic hemodilution [ANH]) for each case was recorded. The rates of performing appropriate pre-transfusion assessments during and after surgery were calculated and compared between the 2 years.

**Results:**

We reviewed 270,421 cases from nine hospitals. A total of 15,739 patients received blood transfusions during the perioperative period. The rates of intraoperative allogeneic transfusion (74.8% vs. 49.9%, *p* <  0.001) and postoperative transfusion (51.9% vs. 44.2%, *p* <  0.001) both decreased from 2007 to 2011; the rates of appropriate assessment increased significantly during (63.0% vs. 78.0%, *p* <  0.001) and after surgery (70.6% vs. 78.4%, *p* <  0.001). The number of patients who received cell salvage or ANH was higher in 2011 (27.6% cell salvage; 9.3% ANH) than in 2007 (6.3% cell salvage; 0.1% ANH).

**Conclusion:**

Continuing education and implementation of updated PoBM guidelines resulted in significant improvements in the quality of blood transfusion management in hospitals in Zhejiang, China.

**Electronic supplementary material:**

The online version of this article (10.1186/s12913-018-3673-x) contains supplementary material, which is available to authorized users.

## Background

Blood shortages are a growing problem in China [1–3]. The need for blood transfusions has steadily increased due to an increasing number of patients undergoing surgical care [4]; inappropriate transfusion practices have also increased [5, 6]. Blood is a valuable and vital resource: it should be cautiously managed and used only when truly needed [7–9]. To improve the management of blood resources and clinical transfusion practices, the Ministry of Health of the People’s Republic of China issued *Guidelines for Clinical Use of Blood* in 2000 [10].

In 2007, the Clinical Anesthesia Quality Control Center of Zhejiang Province (AQCZ) initiated a retrospective multicenter assessment of blood transfusions to surgery patients in 9 select hospitals across Zhejiang Province [11]. Of the 19,102 perioperative transfusions reviewed, 44.1% were performed without testing hemoglobin (Hb) or other necessary laboratory parameters prior to administration of the blood. Instead, decisions regarding blood transfusions were based solely on the clinical judgments of the anesthesiologists or surgeons. The rate of inappropriate red blood cell (RBC) transfusions among patients with complete transfusion records was 39.2% [11]. These unsatisfactory findings urged the health administration and hospital management committees to update perioperative blood management (PoBM) guidelines. In 2008, hospital administrators in Zhejiang Province were trained on the new PoBM guidelines through a series of lectures and conferences. In 2009, the PoBM guidelines were introduced to all hospitals in Zhejiang Province as a key factor to ensure patient safety and healthcare quality [12]. By 2010, the importance of the updated guidelines was promoted with a campaign of posters, educational programs, training courses, and site evaluations, and compliance with the guidelines became a decisive criterion for hospital service and quality ranking [13, 14].

In this study, we aimed to determine if efforts made since the introduction of the new PoBM guidelines led to changes in clinical blood management and patient safety. Specifically, we examined whether blood transfusion quality improved after the 3-year education and implementation period in the hospitals in Zhejiang. The AQCZ initiated a second retrospective investigation in 2012. This report compares data collected on perioperative blood transfusions that occurred in 2007 and 2011.

## Methods

### Data collection

The province of Zhejiang is a mid-size province located in the southeast corner of China. It has a total population of approximately 55 million people, and there are 31 major hospitals in Zhejiang Province. To maintain regional balance, we randomly selected 3 tertiary hospitals in each of the three regions in Zhejiang, i.e. south, north, and central regions to include a total of nine hospitals in the study. Specifically, we drew three cards from a deck of poker cards matching to the number of hospital in each region. Each of the selected hospitals has more than 1000 beds and each performs more than 10,000 surgical procedures annually. We conducted a retrospective, multicenter, cross-sectional case review study on these 9 hospitals.

### Inclusion and exclusion criteria

The adult (i.e., ≥ 18 years old) hospitalized patients who underwent surgery during either of 2 time periods: January 1 to December 31, 2007 and January 1 to December 31, 2011 were included. Among these cases, those who received blood transfusion were selected for medical chart review.

Patient under 19 years of age were excluded. Cases without sufficient information on demographics and blood transfusion information were also excluded. The study protocol was reviewed and approved by the Ethics Review Committee at the Second Affiliated Hospital of Zhejiang University.

A structured survey form was designed by the study committee members in order to collect critical data on the quality of blood management. The study committee members, including one senior anesthesiologist(MY), three junior anesthesiologists (YYY, and other two), and three hospital quality control officers representing the AQCZ, had previously completed in-depth training on all study definitions.

Due to a lack of electronic records in the hospitals, the study committee members reviewed medical charts at each hospital and manually entered data into a computer. The data entry period lasted 23 months: from June 2012 to May 2014. To ensure that data entry was valid, consistent, and followed the study protocol, a portion of medical cases were selected and reviewed by all committee members. Specifically, computer generated digits were used for us to randomly select 70 medical among all hospital cases on each month. As a result, a total of 1600 cases have been reviewed to ensure quality of data collection.

In each hospital, we reviewed cases of patients who received a blood transfusion during or after surgical care. For each case, we collected the patient’s demographic information, including age, sex, weight, days of hospitalization, type of admission (i.e., scheduled or emergency), past medical history, and American Society of Anesthesiology (ASA) grade prior to surgery. We also recorded the type of surgical procedure, duration of operation, any serious perioperative complications, and volume of blood loss during the surgery. To assess the quality of blood management, the results of all available blood laboratory tests were recorded over a period of 10 days, i.e., 7 days prior to operation and 3 days after the operation, along with the volumes of allogeneic RBC transfusions during the surgery. We recorded the Hb level before and after the transfusion, and, for autologous RBC transfusion, we recorded intraoperative and postoperative cell salvage and acute normovolemic hemodilution (ANH). Intra-hospital mortality rate after the surgery and the length of hospital stay were also collected for each case (Additional file 1: Figure S1).

We defined preoperative Hb concentration as the last Hb measurement before the index surgery. Preoperative anemia was defined according to the Chinese Medicine Association’s sex-based criteria [15]: Hb concentration less than 11 g/dl for women and Hb concentration less than 12 g/dl for men. Pre-transfusion Hb concentration was defined as the last Hb measurement before the transfusion. Post-transfusion intraoperative Hb concentration was defined as the last Hb measurement before the patient was sent to the ward; post-transfusion postoperative Hb concentration was defined as the last Hb measurement within 3 days of the transfusion.

### Appropriateness of transfusion

We used the national blood transfusion guidelines, recommendations from the World Health Organization’s *The Clinical Use of Blood* [16] and the American Association of Blood Banks’ *Clinical Practice Guideline* [17], and results of clinical research [18–20] to define the appropriateness of transfusions (Table 1).

**Table 1:** Definition of appropriateness assessment

With Lab Test / Appropriate: • Pre-transfusion Hb concentration < 7 g/dl; • Pre-transfusion Hb concentration 7–8 g/dl, and preoperative chronic anemia; • Pre-transfusion Hb concentration 7–10 g/dl, and age ≥ 65 years or ASA grade in III-V or blood loss ≥1000 ml; • Pre-transfusion Hb concentration 7–10 g/dl, and renal failure or history of cardiovascular disease, hematencephalon, lungs injury or undergone neurosurgical operations, orthopedic operations or cardiovascular operations;	
With Lab Test / Inappropriate: • Pre-transfusion Hb concentration 7–10 g/dl, without above mentioned comorbidities, or pre-transfusion Hb concentration > 10 g/dl.	
Without Lab Test: /Inappropriate Lack of Hb and other testing records before and after allogeneic RBC transfusion.	

### Statistical analysis

Statistical analyses were performed with SPSS 17.0 software (Chicago, IL). Variables were described as number and percentage, mean and standard deviation, and median and interquartile range, in the case of a non-normal distribution. Continuous data were analyzed by Student’s *t*-test or by one-way analysis of variance, as appropriate. Dichotomous variables were analyzed by the chi-square test. A *p*-value less than 0.05 was considered statistically significant.

## Results

**Fig. 1:**
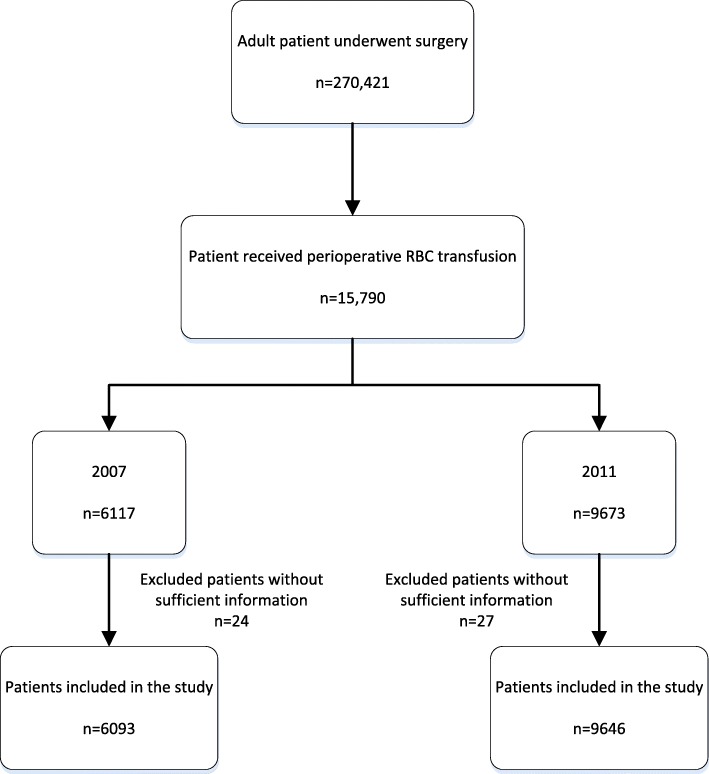
The flowchart of data collection

We identified 270,421 adult inpatients who underwent surgery at nine hospitals during the 2 study periods. Among them, 15,790 patients received RBC transfusions intraoperatively or within 3 days after surgery. For patients who underwent multiple surgeries related to the same health problem, we only included the data for the patient’s first surgery. We excluded 51 (0.3%) patients whose medical records did not have sufficient information about baseline characteristics. The final analysis included 15,739 patients (Fig. 1).

Patient demographics, preoperative conditions, and postoperative management are shown in Table 2. Compared with patients who received perioperative RBC transfusions in 2007, patients who received blood transfusions in 2011 were older, had a higher body weight, and were more likely to be female and have a high ASA status (*p* <  0.001); transfusion recipients in 2011 also had a higher prevalence of hypertension (*p* <  0.001) and diabetes (*p* = 0.002). Transfusions in 2011 were less likely than transfusions in 2007 to be associated with emergent cases (*p* <  0.001). Furthermore, patients in 2011 were more likely to be transferred to an intensive care unit and have a longer hospitalization after surgery (*p* = 0.002); however, they had a shorter overall hospital stay (*p* <  0.001) and a lower incidence of in-hospital death (*p* = 0.037).

**Table 2:** Demographics

	2007	2011	
	*n* = 6093	*n* = 9646	*p* value
Age (Year, mean ± SD)	50.7 ± 17.1	53.7 ± 16.5	< 0.001
Weight (Kg, mean ± SD)	59.0 ± 10.5	59.9 ± 10.6	< 0.001
Female (Count; %)	2531; 41.5%	4554; 47.2%	< 0.001
ASA (Count; %)			
I	952; 15.6%	1299; 13.5%	< 0.001
II	3292; 54.0%	5516; 57.2%	< 0.001
III	1462; 24.0%	2050; 21.3%	< 0.001
IV-V	387; 6.4%	781; 8.1%	< 0.001
General Anaesthesia (Count; %)	5388; 88.4%	8750; 90.7%	< 0.001
Preoperative Anemia (Count; %)	2701; 44.3%	3831; 39.7%	< 0.001
Emergency Case (Count; %)	1939; 31.8%	2456; 25.5%	< 0.001
Surgery subspeciality (Count; %)			< 0.001
General	1610; 26.4%	1935; 20.1%	< 0.001
Cardiovascular	310; 5.1%	647; 6.7%	< 0.001
Orthopedic	1954; 32.1%	3766; 39.0%	< 0.001
Neurosurgical	749; 12.3%	1305; 13.5%	0.013
Other^a^	1470; 24.1%	1993; 20.7%	< 0.001
Duration of Surgery (Min, mean ± SD)	180 ± 93.6	177 ± 97.2	0.096
Post-OR ICU Stay (%; Day; mean ± SD)	25%; 5 ± 7.0	27%; 6 ± 9.8	0.002
Hospital Stay (Day; mean ± SD)	27 ± 24.4	24 ± 19.9	< 0.001
In-hospital death (Count; %)	134; 2.2%	167; 1.7%	0.037
Comorbidities (Count; %)			
Hypertension	713; 11.7%	1931; 20%	< 0.001
Cardiovascular problems	212; 3.5%	366; 3.8%	0.164
Chronic Obstructive Pulmonary Disease	115; 1.9%	188; 1.9%	0.417
Diabetes	284; 4.7%	558; 5.8%	0.002

^a^Gynaecological, urological, otolaryngological, plastic, or thoracic

### Cross-sectional analysis

The rate for allogeneic blood transfusion was reduced in 2011 than in 2007 (4.7% vs. 5.4%, *p* <  0.001) (Table 3). During surgery, a smaller portion of patients received allogeneic blood transfusions in 2011 than in 2007 (49.9% vs. 74.8%, *p* <  0.001), with a relatively smaller amount of mean allogeneic RBC use in 2011 than in 2007 (671 ± 472 ml vs. 702 ± 503 ml, *p* <  0.001). The moderate blood transfusion rate in 2011 was achieved with older patients and in more critical cases and emergent situations than in 2007 (Table 2). The pre-transfusion Hb test rate was higher in 2011 than in 2007 (67.5% vs. 37.0%, *p* <  0.001).

**Table 3:** Blood transfusion data between 2007 and 2011

	2007	2011	*P* value
Patient receiving surgery (N)	111,655	158,766	
Patient receiving blood transfusion during hospital care (N; %)	6093; 5.4%	9646; 6.1%	< 0.001
Patient receiving perioperative allogeneic RBC transfusion (N; %)	6000; 5.4%	7468; 4.7%	< 0.001
Intra-operative			
Intra-operative blood loss (ml, mean ± SD)	873 ± 1000	722 ± 812	< 0.001
Intra-operative blood transfusion (N; %)	4560; 74.8%	4812; 49.9%	< 0.001
Intra-operative blood transfusion (Volume in ml, mean ± SD)	702 ± 503	671 ± 472	< 0.001
Pre-transfusion haemoglobin test (N; %)	1688; 37.0%	3250; 67.5%	< 0.001
Transfusion haemoglobin (g/dl, mean ± SD)	93.1 ± 26.7	89.1 ± 24.3	0.002
Pre-transfusion Hct (%, mean ± SD)	28.5 ± 8.1	26.9 ± 7.0	< 0.001
Appropriateness (N; %)	1063; 63%	2535; 78%	< 0.001
Post-operative			
Post-operative blood loss (ml, mean ± SD)	500 ± 609	491 ± 621	0.46
Post-operative blood transfusion (N; %)	3164; 51.9%	4268; 44.2%	< 0.001
Post-operative blood transfusion (Volume in ml, mean ± SD)	712 ± 562	632 ± 517	< 0.001
Pre-transfusion haemoglobin test (N; %)	1898; 60.0%	3042; 71.3%	< 0.001
Transfusion haemoglobin (g/dl, mean ± SD)	89.9 ± 22.5	85.4 ± 20.2	< 0.001
Pre-transfusion Hct (%, mean ± SD)	26.8 ± 6.7	25.5 ± 6.0	< 0.001
Appropriateness (N; %)	1340; 70.6%	2386; 78.4%	< 0.001
Cell salvage or ANH			
Acute normovolemic hemodilution (N; %)	3; 0.1%	894; 9.3%	< 0.001
Intra-operative cell salvage (N; %)	383; 6.3%	2665; 27.6%	< 0.001
Average volume of cell salvage per patient intraoperative (ml)	63	172	
Post-operative cell salvage (N; %)	17; 0.3%	41; 0.4%	< 0.001

Improved blood management was also observed during the postoperative phase. Simply, a smaller portion of patients received postoperative blood transfusions in 2011 than in 2007 (51.9% vs. 44.3%, *p* <  0.001), with a relatively smaller mean volume of allogeneic RBCs in 2011 than in 2007 (632 ± 517 ml vs. 712 ± 562 ml, *p* <  0.001). The rate of pre-transfusion Hb test was also significantly higher in 2011 than in 2007 (71.3% vs. 60.0%, *p* <  0.001) (Table 3).

The number of patients receiving intraoperative cell salvage or ANH significantly increased in 2011. A total of 2665 (27.6%) patients in 2011 received cell salvage, compared with 383 (6.3%) patients in 2007 (*p* <  0.001); 894 (9.3%) patients in 2011 received ANH, compared with only 3 (0.1%) patients in 2007 (*p* <  0.001). In total, 2665 cell salvage patients received 1,282,623 ml and 894 ANH patients received 334,497 ml intraoperatively in 2011; 383 cell salvage patients received 295,979 ml and 3 ANH patients received 1350 ml intraoperatively in 2007. Postoperatively, the number of patients receiving cell salvage was small and only occurred in some hospitals (41 patients from four hospitals in 2011, 17 patients from six hospitals in 2007). Therefore, we did not perform statistical analysis of postoperative cell salvage data (Table 3).

### Appropriateness evaluation

We checked medical records for laboratory results of Hb and other blood tests before and after transfusions. The proportion of intraoperative blood transfusions without appropriate assessment in 2011 was significantly lower than in 2007 (intraoperatively: 32.5% vs. 63.0% [*p* <  0.001]; postoperatively: 28.7% vs. 40.0% [*p* <  0.001]). We evaluated the appropriateness of the transfusions according to composite criteria, adjusting for surgical procedure, age, sex, ASA status, preoperative anemia, and emergency status. The rate of appropriate intraoperative transfusions increased from 62.9% in 2007 to 78.0% in 2011 (*p* <  0.001); postoperatively, the rate of appropriate transfusions increased from 70.6 to 78.4% (*p* <  0.001) (Table 3).

### Intraoperative and postoperative comparison

In China, the anesthesiologist takes the primary decision-making role regarding blood transfusions during surgery; after surgery, the chief surgeon makes these decisions. To investigate whether anesthesiologists and surgeons present different behaviors regarding blood management, we investigated differences in the intraoperative and postoperative periods. The rate of Hb testing (marginal means of 2007 and 2011) before intraoperative blood transfusion was 52.7% and the rate of testing in the postoperative phase was 66.5% (*p* <  0.001). When this number was assessed according to year, a dramatic improvement was observed from 2007 to 2011 during the intraoperative phase (37.0 to 67.5%) and a smaller improvement was seen during the postoperative phase (60.0 to 71.3%) (Table 3).

## Discussion

We noted important and significant impacts for patients in Zhejiang who underwent surgery after the implementation of updated PoBM guidelines. In 2007, a considerable number of perioperative transfusions were probably performed on the basis of subjective assessments by medical practitioners. Furthermore, only 383 (6.3%) patients in our survey received cell salvage or ANH. Our data showed that more ASA IV-V patients were accepted operation in 2011. In addition, a larger percentage of orthopedic and cardiovascular cases were performed in the nine survey hospitals in 2011, which indicate that more critical patients were treated in 2011 compared to 2007. However, in 2011, the allogeneic RBC transfusion rate on patient was significantly reduced. Significantly more perioperative blood transfusions in 2011 were given based on patients’ Hb levels and other objective laboratory reports. The rates of appropriate intraoperative and postoperative blood transfusions both increased from 2007 to 2011. Several factors that may have contributed to these improvements in blood management are presented below.

### Guideline development and implementation

The Zhejiang PoBM strategies were created based on a national version of guideline (*Guidelines for Clinical Use of Blood* [21]) issued by the State Public Health Department. With its patient-centered philosophy, the updated PoBM guidelines are best implemented within the framework of an organized and recognized program [22]. Guidelines for Clinical Use of Blood proposed a restrictive transfusion strategy and recommended transfusion at Hb concentrations of 7 g/dl or less; transfusions are unnecessary at Hb concentrations of 10 g/dl or more. Between the 2 thresholds, there are no absolute, universal recommendations in existing guidelines, and transfusion-related decisions should be considered according to clinical factors, such as cardiopulmonary function, the severity of anemia, basic metabolic rate, and age. In this context, inappropriate blood transfusions are sometimes ordered. In this study, we assessed the appropriateness of transfusions on the basis of whether surgeons and anesthesiologists considered clinical factors before ordering blood transfusions for patients.

Several problems existed among the hospitals in Zhejiang prior to the implementation of the new guidelines. Many clinicians permitted blood transfusions when Hb levels were 10 g/dl or higher in 2007, which resulted in a high number of allogeneic RBC transfusions: many of these were unnecessary. Except in special emergency cases, a patient’s Hb level should be measured to determine the need for transfusion. According to our findings, after the implementation of updated PoBM guidelines*,* the rate of inappropriate blood transfusions decreased from 2007 to 2011.

From 2008 to 2010, a series of educational programs and interventions were conducted in Zhejiang Province to actively promote the implementation of the new PoBM guidelines. Specifically, the AQCZ organized and presented more than 30 continuing education courses between December 2009 and September 2011. Surgeons, anesthesiologists, and transfusion physicians from tertiary and secondary hospitals across Zhejiang Province attended these courses, and the healthcare providers were offered multiple opportunities to interact and discuss issues related to blood management.

Hospitals in Zhejiang were expected to comply with the following regulations: a clinical transfusion management committee must be established at the hospital level; blood transfusions must be monitored in accordance with the PoBM guidelines and evaluations must be performed before and after transfusions to ensure the effective and safe use of blood; strategies must be developed to promote blood conservation techniques including the use of cell salvage or ANH; and, necessary education and training courses must be developed for physicians and surgeons to ensure that knowledge is translated to clinical practice [14]. These high-profile initiatives not only positioned compliance with PoBM guidelines as a major public health issue on the administrative agenda but also resulted in unprecedented improvements in blood transfusion practices of hospitals in Zhejiang.

Since 2008, all nine hospitals included in our study established clinical transfusion management committees and subsequently implemented the new PoBM guidelines. Seven hospitals regularly provide formal training in transfusion practices to their staff, including surgeons, physicians, anesthesiologists, and nurses.

The proportion of cell salvage or ANH increased from 2007 to 2011. The nine hospitals in this study reported large numbers of cases involving intraoperative cell salvage and ANH in 2011, which possibly explains the significant reduction in allogeneic RBC use [23–25]. Postoperative autologous blood transfusion, reinfusion of RBC harvested from the drainage system, has been reported in the other studies that can contribute to the reduction in allogeneic blood transfusion [26, 27]. In our study, the postoperative RBC cell salvage was carried out only on two hospitals, giving a total of 28 cases. We suggest to promote the postoperative cell salvage technique to more hospitals and we hope to see further improvements in future.

### Role of primary healthcare providers

Typically in China, anesthesiologists are responsible for intraoperative transfusion decisions while surgeons more often make decisions in the postoperative period. In this study, we separately examined the rates of appropriate intraoperative and postoperative blood transfusions and found that both professional groups showed significant improvements in blood transfusion management. We observed a more dramatic improvement during the intraoperative phase than during the postoperative phase. This evidence suggested that anesthesiologists in Zhejiang are, perhaps, more prepared to update their management strategy than their surgery partners when considering blood transfusions in this few years. Nevertheless, there were still 32.5% intraoperative and 28.7% postoperative transfusion occurred in 2011 without laboratory test. Completely eliminating those undesirable blood transfusion cases can be challenged for both anesthesiologists and surgeons during different phases of patient care. We believe that greater efforts should still be aimed at anesthesiologists and surgeons, as they are the primary care delivering and the decision-making person for blood transfusion.

### Improving awareness and education among patients and the public

In addition to educating healthcare providers, effective PoBM requires public involvement and patient support. Patient engagement and understanding is essential to the successful implementation of blood management guidelines. In addition to the delivery of educational programs to healthcare providers, the health authority also completed a series of patient awareness events. Critical information on the safe blood management was published in the local newspapers and community websites to educate patients and their family members [28, 29]. These efforts prompted a change in people’s mindset towards safe blood management. New concepts such as “transfusion-free surgical hospitals” and “autologous transfusion” have been graduated accepted by the community. A piece of evidence reflecting of this change was that that more patients in 2011 sign consent to accept autologous blood transfusion than in 2007, which encourages us to further our efforts to educate people in the community to engage with safe blood management.

### Strengths and limitations

This is the first in a series of reports on blood management practices in China using large-scale patient-based data collected from multiple hospitals. All hospitals included in our study are general hospitals, which provide services to people with serious health problems and are considered the primary targets for quality control in blood management. The data collected from these hospitals and the results presented in this paper accurately represent the blood management situation in the Zhejiang Province of China.

There are several important limitations to this study. First, we only audited blood transfusion quality for patients who underwent surgery; therefore, caution is needed when applying our findings to non-surgery scenarios. Second, data collected from selected hospitals in Zhejiang may represent the majority of blood transfusion cases, but it does not describe the transfusion practices in specialty hospitals such as women and children’s hospitals, dental hospitals, oncology centers, and rehabilitation hospitals. Future research to describe the status of PoBM in China should include data from a wider range of hospitals and cases of non-surgery patients. Third, we reported here the data up to 2011. Data of later years are still collected. We will continue to work on these data and understand the impact of PoBM education and initiatives on the patient safety.

## Conclusion

Our findings highlight the significant improvements in PoBM that resulted from the implementation of updated transfusion and PoBM guidelines introduced by the health department in Zhejiang Province. This successful implementation prompted China’s Ministry of Health to include PoBM-specific requirements in the national accreditation standards for tertiary general hospitals in 2011. Ongoing improvements have led to greater compliance with PoBM guidelines, although the data is yet to be analyzed.

## Additional file

Additional file 1: **Figure S1.** The information of structured survey. Description of data: The indexes of demographic information, operation information and blood management information we collected. (DOCX 15 kb)

## Abbreviations

ANH: Acute normovolemic hemodilution
AQCZ: the Clinical Anesthesia Quality Control Center of Zhejiang Province
ASA: American Society of Anesthesiology
Hb: Hemoglobin
PoBM: Perioperative blood management
RBC: Red blood cell
