# Supplementary material for: Improvements in blood transfusion management: cross-sectional data analysis from nine hospitals in Zhejiang, China
Source: BMC Health Serv Res. 2018 Nov 14;18:856. doi: 10.1186/s12913-018-3673-x (PMC6237039; doi:10.1186/s12913-018-3673-x)
Supplement: Supplementary file 1 — Figure S1. The information of structured survey. Description of data: The indexes of demographic information, operation information and blood management information we collected. (DOCX 15 kb) [file 12913_2018_3673_MOESM1_ESM.docx]

Figure S1 The information of structured survey.

| Demographic information |  |
| --- | --- |
|  | Age |
|  | Sex |
|  | Weight |
|  | Height |
|  | ASA grade |
|  | Type of admission |
|  | Past medical history |
| Operation information |  |
|  | Time |
|  | Type of surgical procedure |
|  | Duration of operation |
|  | Type of anesthesia |
|  | Blood loss |
|  | Post-OR ICU Stay |
|  | Hospital Stay |
|  | Serious perioperative complications |
|  | Outcomes after surgery |
| Blood management information |  |
| Intraoperative | Preoperative Hb tests level |
|  | Volumes of RBC transfusion |
|  | Hb level before and after transfusion |
|  | Cell salvage |
|  | Acute normovolemic hemodilution |
| Postoperative | Volumes of RBC transfusion |
|  | Hb level before and after transfusion |
|  | Hb level before and after transfusion |
|  | Acute normovolemic hemodilution |
|  |  |
